# Optimising survivorship post-BMT: healthcare professionals’ perceptions of long-term care

**DOI:** 10.1007/s00277-025-06398-8

**Published:** 2025-05-17

**Authors:** Gemma McErlean, Christine Ashley, Anisha Pradhan, Vanessa Yenson, Ian Kerridge, Elizabeth Halcomb

**Affiliations:** 1 School of Nursing, Faculty of Science, Medicine & Health, University of Wollongong, Northfields Ave Wollongong, NSW 2522 Australia; 2 Health Innovations Research Centre, Faculty of Science, Medicine & Health, University of Wollongong, Northfields Ave Wollongong, NSW 2522 Australia; 3 Centre for Research in Nursing and Health, St George Hospital, Kogarah, NSW Australia; 4 The Centre for Improving Palliative, Aged and Chronic Care through Clinical Research and Translation (IMPACCT), Faculty of Health, University of Technology Sydney, Sydney, Australia; 5 Royal North Shore Hospital and Sydney Health Ethics, University of Sydney, Sydney, NSW Australia

**Keywords:** Blood and marrow transplant, Health professional role, Communication, Data access, Survivorship, Models of care, Shared care

## Abstract

Survivors who have received a blood or marrow transplant (BMT) require life-long follow-up care and support, however evidence-based assessments of long-term models of care are scarce. This qualitative descriptive study explored the perspectives of blood and marrow transplant (BMT) specialist nurses and physicians, and General Practitioners (GPs) regarding the long-term management and follow-up care of BMT survivors. Semi-structured online interviews were conducted with thirteen purposefully selected BMT Physicians (*n* = 4), Advanced Practice Nurses (APNs) (*n* = 6) and GPs (*n* = 3), experienced in providing long-term care to BMT survivors. Data were analysed using thematic analysis. Both specialist and community-based practitioners identified deficiencies in models of care delivery and in the organisation of long-term care for BMT survivors, particularly in relation to communication, transition and personalisation of care. Two themes were identified that explored (1) Efficiency and quality of current care provision for BMT survivors and (2) Characteristics of optimal care. All participants recognised the need for flexible, patient-centred models of shared care that bring together hospital and community-based healthcare professionals in providing optimal care to BMT survivors. The growing population of survivors of BMT requires an urgent re-evaluation of healthcare models to address their complex long-term care needs. This will require a well-trained primary care-based workforce supported by collaborative relationships with specialist centres and easy access to essential information. Current approaches to post-BMT care that limit provision of care to specialist BMT services are unsustainable, inefficient, and do not support the transition of patients from tertiary to community healthcare services.

## Introduction

Despite growing interest in cancer survivorship care internationally and the evolving role of community-based health professionals in providing some long-term care, evidence-based descriptions and assessments of various models of care are scarce [1, 2]. In particular, survivors of blood and marrow transplant (BMT) often require complex life-long care, placing significant burdens on patients, their carers, and the entire health system [3]. In an Australian study Dyer [4] estimated that due to the range of morbidities experienced by survivors of BMT, allogeneic survivors may require up to 34 assessments or consultations involving six specialties each year.

Historically, BMT specialist centres have assumed responsibility for providing guideline-based, long-term follow-up care [3]. However, this is unsustainable, inefficient and can result in poor quality care as transplant clinicians are overburdened with a growing survivor population and a plateauing workforce [5, 6]. Additionally, clinicians in these acute settings often lack training or expertise in managing the long-term issues experienced by survivors of BMT. These long-term issues may include providing health counselling, health promotion and preventive care advice, and advising on sexual dysfunction or psychosocial care [4, 7].

In Australia, autologous and allogeneic Blood and Marrow Transplantation (BMT) services are provided by 41 hospitals located in major urban centres across the country. These centres perform almost 1,800 transplants annually [8]. Among these, five hospitals also offer CAR-T cell therapy. Each of these BMT centres provides long-term follow-up care for survivors. Although there are minor structural variations in the approach to long-term BMT follow-up, generally, care is coordinated and delivered within the transplant service itself.

Some Australian BMT centres employed BMT Advanced Practice Nurses to co-ordinate and manage long-term survivors’ care, which has increased adherence with long-term follow-up protocols. However, this model is not considered sustainable [9, 10] due to its reliance on specialised resources. Additionally, it continues to place hospitals as the centre of post-BMT care despite the fact that many survivors no longer require inpatient care and often live long distances from specialist centres [11].

Shared care models, which involve coordination between specialist and primary care providers, represent a promising alternative approach. General Practitioners (GPs) and general practice nurses (GPNs) offer several advantages for BMT survivors’ long-term care: they are located closer to survivors’ homes, often have existing relationships with survivors of BMT and their families, and have expertise in screening and prevention, health promotion, health education and infection prevention through vaccination [12]. However, before implementing such models, it is essential to understand the perspectives of all healthcare professionals involved to ensure that any new model is feasible, sustainable, meets patient and health service needs, promotes best-practice care and optimises health outcomes.

While the contribution of primary care to survivorship following treatment of solid cancers has been increasingly investigated in recent years, far less research has examined the views, capability, and confidence of healthcare professionals in shared management of long-term BMT survivors As a critical first step towards re-engineering follow-up care structures, this paper aims to explore healthcare professionals’ (HCPs) perspectives on current and potential models of shared care for BMT survivors.

## Methods

### Study design

This was a qualitative descriptive study using semi-structured interviews with clinicians who have provided care for survivors of BMT [13]. This study method enabled in-depth exploration of participants’ experiences of and view towards optimal long-term management and follow-up care. The Consolidated Criteria for Reporting Qualitative Studies (COREQ) [14] checklist was used to guide reporting.

### Participants

HCPs were included if they had recent experience in providing care to survivors of BMT. All participants were currently registered in with the Australian Health Practitioner Regulation Agency (AHPRA). For nursing professionals, a minimum of one year’s experience in caring for BMT survivors was required. HCPs who exclusively provided inpatient care in the pre- or early post-BMT period were excluded from the study.

### Recruitment

Targeted recruitment of GPs took place via purposive sampling, with direct contact following identification of their involvement in care from BMT databases at selected haematology departments in NSW. Potential participants were contacted by the researchers, provided with information about the study and invited to participate. Those consenting to participate were then contacted and interviews organised at a mutually agreed time.

Recruitment of GPs, APNs, and Specialist Haematologists employed both purposive and snowball sampling. Strategies included disseminating study information over a period of two months through primary health networks and other professional organisations and social media. Information about the study included the researcher’s contact information. All potential participants were sent information packages and consent forms. Interviews were organised at a mutually agreed time.

Efforts were made to select participants to include representation from those working in metropolitan, regional and rural areas.

### Data collection

Thirteen semi-structured phone interviews were conducted by two members of the research team (GM and AP). The interviews followed a semi-structured interview schedule designed by the researchers based on the literature and their clinical experience. Probes and prompts were used to further explore ideas raised. The interviews varied from 21 to 60 min in length. Topics included participants knowledge and experiences of caring for BMT survivors, attitudes towards long-term survivorship, views on shared follow-up care and transitioning from BMT centres to primary care, and finally, barriers and facilitators to providing long-term care within both hospital environments and community settings. All interviews were digitally recorded and transcribed verbatim by a professional transcription company. Interviews were undertaken until data saturation was achieved, that is, the interviewer was perceiving that no new information was forthcoming in subsequent interviews.

### Data analysis

Transcripts were imported into NVivo™ (NVivo software, 2012) to facilitate data analysis. Identifying data were removed prior to analysis and pseudonyms were used to protect the identity of participants and/or their workplaces.

Data were analysed using Braun and Clarke’s reflexive thematic analysis approach [15]. This six-phase method includes; familiarisation with the data, generating initial codes, searching for themes, reviewing themes, defining and naming themes, producing the report [15]. Two researchers independently coded the first three transcripts using an open coding approach, generating initial codes directly from the data. The research team then met to discuss these into initial codes and develop a preliminary coding framework. This framework was applied to subsequent transcripts, with new codes added inductively as they emerged. Codes were then organized into potential themes that captured important patterns relevant to our research questions. These initial themes were iteratively reviewed and refined through team discussion until consensus was reached on the final thematic structure, ensuring themes worked both in relation to the coded extracts and the entire dataset.

### Ethics

The Human Research Ethics Committees of the South Western Sydney Local Health District (Approval No. 2022/ETH01503) approved this project. All participants were provided with study information and advised of their rights regarding participation, and their right to withdraw at any time. Written consent to participate was provided by all participants.

### Rigour

Lincoln and Guba’s [16] criteria of credibility, dependability, confirmability, and transferability were used to demonstrate rigour. Checking of transcripts and researcher consensus demonstrated credibility. Dependability was demonstrated via documentation of decisions around study conduct. Meetings of the research team to discuss and reflect on the study support confirmability. Transferability was demonstrated by providing detailed evidence which allow evaluation of the application of findings to different contexts.

## Results

Thirteen interviews were conducted, comprising APNs (*n* = 6), BMT physicians (*n* = 4) and GPs (*n* = 3). Participants spoke of their perceptions and experiences of caring for BMT survivors, and possible solutions to providing sustainable long-term care to this growing patient population. Two themes that explored models of care delivery were identified: (1) Current care provision for BMT survivors and (2) Characteristics of optimal care.

### Current care provision for BMT survivors

Diverse current pathways to providing long-term follow-up following BMT were described. The different approaches used reflected differences in referral processes, location, staffing and resources, ‘case-mix’ (the type and number of transplants performed, specifically regarding autologous or allogeneic transplant), and the differing roles of health professionals across individual services.

*“It’s basically just me slotting people into my existing clinics for the purposes of long-term follow-up and survivorship sort of clinic.… we’re not really equipped to do the one-stop-shop that a lot of bigger centres…do.”* (BMT Physician #1).

*“We don’t have a long-term follow up provision. We give them a copy of the guidelines… they see their specialist*,* there’s no nurses involved”* (APN #1).

*“…they’ll come to us for one to two years for their maintenance treatment. They will then be linked with the haematology care coordinator who… is kind of the go-to person. And then obviously they still see the doctor in clinic during that maintenance period as well. So they tend to be pretty much followed up for at least two years.”* (APN #5).

Long-term follow-up for survivors of allogeneic transplant was noted to be more complex than that required for survivors of autologous transplant, with care provided for longer in the specialist centre where issues can be addressed more readily:

*“Yes*,* of course if they [allogenic patients] relapse*,* for example*,* or if they get much worse graft versus host disease*,* yes. And it’s much less known what’s going to happen. It’s much less protocol driven and I mean it’s much more dependent on a multitude of different factors that can change”*(*APN* #3).

Geographic location and availability of a referring team interested in shared care was reported to work well in some centres:

*“Because we do transplant patients from rural areas*,* and you just can’t demand that patients come back regularly. So*,* those patients actually go to shared care…*,* it’s actually shared care short-term…as well as long-term follow up. And that can work really well*,* depending upon how keen the referring team is to do shared care” (BMT Physician #3).*

*“…if the patient requires an allogeneic stem cell transplant*,* and that person [doctor] is not a transplanter*,* then they would be referred on to one of the haematologists with an interest in transplant… sometimes they keep them for life*,* and sometimes they’ll keep them for five years and then hand them on to me [haematologist interested in long-term BMT follow-up]*,* depending on who it is. ”* (BMT Physician #1).

.

For patients living in rural areas distant from transplant centres, a number of other factors impacted long-term follow-up including availability of haematologists locally, patient choice, and the capacity to provide care locally:

*“I mean some – if they’re from XXX they might go back and do a bit of shared care with their XXX haematologist. That might depend on how far down south they lived. It might depend on how well they knew the haematologist. It might depend on a number of factors. Yes*,* and it might depend on the patient preference…”* (APN #3).

Some participants described the early and ongoing involvement of the GP in the long-term follow-up, with significant variations in their input.

*“We kind of start the formal long-term follow up process mainly at twelve months. The one thing that starts from six months is the revaccination. And we do usually get the GP involved from that point of view” (BMT Physician #2).*

*“…there’s other aspects of the care that we’re happy to manage during the year with instructions for what we’re trying to do. So often*,* not a formal shared care*,* but it might mean that we’re doing three-monthly checks and organising the various tests*,* reviewing tests*,* doing physical checks. ”* (GP #2).

.

However, others expressed concern about the ability of GPs to appropriately manage complicated follow-up regimes.

*“…a lot of the time with haematology patients*,* the team are often happy to return them to our care…. But something like a transplant*,* I think a lot of GPs wouldn’t really necessarily*,* they can manage lots of the general aspects of their care*,* but the super specialist aspects of their care*,* probably not so comfortable about managing”* (GP #2).

Participants also described the importance of nurse roles in providing long-term follow-up, especially in terms of coordinating care.

*“…there are plenty within my service that haven’t seen their primary haematologist [for* long-term follow-up*]*,* they just see me and whichever consultant is allocated to it. But the main discussions that are occurring are with me. And they ring me and we coordinate. And really*,* it’s like a traffic controller”* (APN #1).

*“Yes*,* so with my Late Effects Clinic…mine’s just purely nurse led. Because to be frank*,* if you actually look at what’s required for the late effects*,* a lot of that is potentially nurse driven. It’s very much about health promotion and education*,* just informing people of what’s still required of them after a transplant”* (APN #6).

Some reported how roles may overlap in the provision of long-term follow-up, and how this sometimes resulted in both patients and health professionals being confused about who was responsible for long-term care:

*“But sometimes I feel like what I’m doing is replicating what their GP’s doing*,* to be honest. Occasionally things come up and I think it’s a good reminder for people to do the screening that is due at that time*,* and immunisations*,* and to pick up on little things”* (BMT Physician #1).

*“I often feel like there’s issues of stepping on each other’s turf and offending people. And I think it’s more a focus on what you are. I don’t know whether it’s got to do with money*,* whether it’s got to do with the funding to the hospitals”* (APN #1).

### Characteristics of optimal care

Participants also described what kinds of care could best meet future demands, including their views on sharing care between specialist and regional centres and primary health care providers. *“I think GPs would be able to care for… [patients]if we have a plan.”* (GP #1). However, it was noted that patient characteristics needed to be carefully evaluated as being appropriate for shared care:

*“…it’s a complex thing… I think [it] depends on whether those patients are going to be off medications and completely cured or not. Those who are going to be on medication for the rest of their life*,* they would need to have specialist feedback for the rest of their life. They should have some connection with some specialised tertiary unit for their supervising”* (GP #1).

*“Yes*,* I think you can really select people for whom the GP is the right person to do it. If the GP wants to do it – I mean if they’re comfortable doing it. And you could always set up a relationship with that GP so that they could consult with you. Particularly in regional areas*,* I think that’s more than reasonable… But there are certainly people where it’s much more complicated. If they’re still on immunosuppression and they’ve still got chronic graft versus host disease*,* then those people are probably more tricky. And you could do sort of alternate leap-frogging appointments with a haematologist or GP. Or just haematologist if necessary”* (BMT Physician #1).

*“you’d have to pick your patients [to have a primary care team do long term follow up]. But if the patients are very well and they’ve had minimal complications*,* and they’re young*,* then doing all those long-term follow-up things I think would be quite appropriate for a GP to do them”* (BMT Physician #2*).*

*“I guess you know which ones to keep [under the care of the haematologist]*,* the ones that are profoundly immunosuppressed or the ones that have significant organ dysfunction…choose which ones are likely to need review…” (BMT Physician #4).*

However, shared care was noted to have resource implications and a need for relationships between care providers if it was to be high-quality care.

*“I think shared care is a really good idea*,* it’s possible to do it*,* but I think it requires – to be done properly at least*,* it requires proper planning*,* proper resourcing*,* and really thinking through exactly how you could standardise it and create relationships that enable patients to be cared for appropriately”* (BMT Physician #3).

Importantly, participants consistently noted that for any model of shared care to function effectively, there needed to be appropriate and ongoing communication between and access to specialist advice and health providers, education provided to health professionals so that they remained confident and capable, and guidelines and protocols to assist the primary care team.

*“Well it’s a matter of providing clear instructions to the GP… good communication. Very clear instructions…some clear dot points about what you’d like and ‘if they run into trouble*,* here’s my email or phone number“* (BMT Physician #4).

*“I suppose…. in an ideal world it’s having that post-transplant coordinator*,* that that purely just looks after the post-transplant patients as like a go-to person and this is who they contact if there’s any issues”* (APN #5).

Some GPs described a desire for training to build the capacity in their practice to care for BMT patients long-term, although there were concerns about how this could be provided:

*“That would be a boost if I could get more coaching or more involvement*,* and then I feel that I’m part of the team*,* and I can actually be more caring.… It might be hard for us to attend during working hours. So if there’s something that can be done after hours or in weekends*,* like seminars or meetings*,* where there would be more exchange of information.”* (GP #2).

*“And probably educational activities for GPs and the nurses in medical practice… in our practice*,* some nurses also do a lot of [the]work*,* actually*,* in the background. So when we are seeing patients*,* they get the patient*,* have a chat with the patient…so some education.” (GP #1).*

Some participants, however, were unconvinced that future models should rely on the primary care team given the already stretched system. BMT Physician #3 described how

*“the general practice system in Australia is in crisis. There’s not enough GPs. GPs are struggling… now is a really difficult time for GPs to be taking on additional work.”.* APN #6 also observed the challenges in that *“locally GPs seem to move on*,* they don’t stay in a practice long-term. Patients want to have that one GP. But it’s almost impractical in this day and age because it’s almost like you’ve got to align yourself with a practice*,* not a GP*,* because you can’t rely on that GP being there”.*

Additionally, APN #1 expressed concern at discharging patients too early as they have “*had so many that have relapsed at two years… it’s just a little bit premature*”.

As a consequence of their experience in providing care to patients living in rural/remote areas and in providing post-transplant care during the COVID-19 pandemic, several participants spoke of the potential benefits that telehealth may provide in supporting long-term follow-up across settings and between health professionals:

*“I work in XXX where they have a team care*,* how do you say*,* team discussion where a patient doesn’t go to the hospital*,* they come to the clinic… The patient is with me and the team is there in the hospital.…moving forward*,* that should be the way*,* actually*,* rather than for patients[having] to go to XXX to take… almost three hours [return trip]” (GP #1).*

*“If there’s some other family or multidisciplinary meeting where there’s a discussion about a patient because of multiple problems a lot of people are involved in*,* the GP can be invited. And that is possible too. So that’s as far as technology is concerned.” (GP #3).*

*“The use of My Virtual Care… or Telehealth has definitely helped…I think it’s the accessibility. And I think we’re getting there with the video… I think it’s the ability for everyone to have access to the same service regardless of where they’re being cared for”* (APN #1).

Several participants also spoke of the need for better data collection and easy access to quality data if shared care models for long-term follow-up are to be effective.

*“In this sort of system*,* it’s hard to get data*,* and hard to measure outcomes of people that are at the clinics.… If they’re just continuing to see their normal haematologist*,* it’s not going to be recorded as a long-term survivorship appointment…like the data collection about it is still not standardised it seems”* (BMT Physician #1).

*“So*,* I think there is a general awareness of what’s needed*,* but the challenge is to set it up. To set up data collection processes. To make sure a data manager knows what they’re doing.”* (BMT *Physician* #3).

*“I’ve got two-year data. And then if they stay within the hospital*,* obviously for every year after that I’ll have data. Yeah*,* but once they’ve returned back to their treating centres*,* it’s sort of hand the baton. And so from a survivorship point of view*,* I don’t know how we harness that and get better data to inform us of what’s going on. Yeah*,* I don’t have an answer…”* (APN #1).

## Discussion

Survivors of BMT require intensive and ongoing healthcare in order to prevent, diagnose and treat the long-term and late effects of transplantation. While this requires involvement of healthcare professionals from many different disciplines working in tertiary institutions and community settings, BMT centres play a crucial role in the coordination and delivery of post-BMT care. As the number of children and adults being transplanted, and the number of patients surviving long-term post-BMT increases it has become increasingly clear that BMT long-term follow-up care provided by specialist centres is not sustainable and requires reconfiguration to meet the needs of the increasing number of people surviving BMT [1, 17]. The results of our study build on results from the limited numbers of other studies reporting on models of care for cancer survivors [1, 17–19] but offer particularly important insights because few of these specifically focus on BMT survivorship, which is particularly complex and places a profound burden on survivors, carers and healthcare systems [4, 19].

While the views and experiences of HCPs in our study were diverse, all participants recognised the need for a flexible shared care model involving haematologists, APNs, specialist centres and primary care services designed to meet individual needs of survivors of BMT. While some participants in this study felt a systems-led approach with established communication channels to transition survivors between hospital and community-based services could be a helpful approach, others noted concerns about the capacity of GPs and nurses to provide quality long term care. Stout [20] found that a simple reliance on the existing knowledge and skills of the primary healthcare workforce to take on the care needs of cancer survivors would not suffice. Rather, there would need to be expert healthcare professionals working in the community who were familiar with existing BMT long-term guidelines [3], and trained in the long-term care of survivors of BMT as well as skilled in providing collaborative care [1]. While our study demonstrated a willingness by GP participants to upskill themselves, study participants were self-selected as having an interest and experience in managing BMT survivors. Interestingly, despite their self-identified ‘experience’ with BMT survivors, they still felt a need to upskill in this area. Australia’s current critical shortage of GPs and nurses and associated staff turnover raises significant barriers to successfully implementing a new program [21, 22]. Gyawali et al. [18] note that similar shortages in Canada led to the development of oncology training programs for GPs to gain skills in caring for cancer survivors. While not specific to BMT, the results of their study are relevant because they demonstrated that flexible training incorporating self-directed or web-based learning options could successfully provide community practitioners with the skills needed to care for survivors of cancer.

Beyond building primary care capacity through education, implementing strategies to reduce the gap between acute hospital and primary care services, such as telehealth-enabled case conferences - can contribute to better team-building and inclusive care planning. The COVID-19 pandemic accelerated telehealth adoption, with systematic reviews highlighting its role in facilitating real-time collaboration between specialists and primary care providers, particularly for follow-up (53% of telehealth applications), triage (22%) and treatment (34%) [23]. In primary care, telehealth has enhanced interprofessional coordination, reduced unnecessary hospital visits, and improved access to specialized services [24]. For BMT survivors, telehealth models like Stanford’s virtual survivorship clinics have successfully connected patients with nurse coordinators and social workers, ensuring continuity during transitions to community care [25].

Additionally, expanding the role of experienced BMT nurses as care co-ordinators during the survivorship period could address systemic fragmentation [32]. Nurse navigators in oncology settings, such as metastatic breast care programs, already demonstrate reduced disparities and improved patient outcomes through complex care coordination and community partnerships [26]. To replicate these successes, community-based BMT nurse outreach roles could bridge hospital and primary care by organizing joint training, facilitating case conferences, and ensuring survivorship care plans are dynamically updated.

Our study participants noted that the collection, storage, availability, and access to clinical data were particularly significant barriers to effective communication between acute and community-based services. This finding is not unique as access to reliable data relating to BMT has been noted to be a recurring problem throughout the world [27]. In Australian jurisdictions, data regulations, including formal legislation and agency-specific policies, are often seen as a barrier to data sharing between acute and community-based care providers [28]. As is the case for many people with multimorbidity and complex conditions, BMT survivors have health data stored across multiple service providers and possibly across jurisdictional borders in Australia. This creates issues with data access, such as specialists being unable to access test results undertaken in regional centres and GPs and community-based nurses being unable to access results from specialist centres with individual medical records systems. While Australia has trialled a system of individual health records, My Health Record, the uptake has been poor due to lack of trust in the systems and the complexity of sharing data between providers [29].

Limitations in data availability also impeded communication between health professionals. However, Nekhlyudov, O’Malley and Hudson [1] advocate that preferred data communication and collection methods will vary due to geographic, insurance and/or financial considerations and aiming for perfect communication scenarios (i.e. easy access via databases) should not impede using other forms of communication. These include using electronic health records, emails, paper documentation, phone calls, face-to-face and virtual consultations and meetings, monitoring apps, and shared survivorship care plans, which could be developed, stored electronically and accessible to all members of the survivorship team [1, 30]. Our findings were consistent with various authors in noting that such communication enhances interprofessional trust and respect, assists in the coordination of care and may avoid duplicating or omitting care [1, 30, 31]. The lack of a coordinated approach to data collection was a key finding in our study, suggesting that this impeded the capacity to provide quality follow-up, measure individual survivor outcomes or undertake research into long-term care outcomes.

## Conclusion

There is an urgent need to better address the complex healthcare needs of the increasing numbers of children, adolescents and adults worldwide surviving BMT and to support them as they transition between specialist to primary healthcare settings. The results of this study make clear that a flexible, patient-centred model for shared care that combines the expertise of both hospital-based and community based medical, nursing and allied health practitioners provides the best model for effective long-term care of survivors of BMT. Such an approach, however, requires a well-trained primary care-based workforce supported through collaborative relationships with specialist centres, collection and access to data, and knowledge and access to policies, procedures and evidence-based guidelines. Further research is required to identify the effectiveness of shared care models for long-term care post-BMT in different settings and its impact on the BMT survivor’s experience and quality of survival.

## Data Availability

The data that support the findings of this study are available from the corresponding author upon reasonable request.
